# Molecular Modeling Studies on the Interactions of Aflatoxin B1 and Its Metabolites with Human Acetylcholinesterase. Part II: Interactions with the Catalytic Anionic Site (CAS)

**DOI:** 10.3390/toxins10100389

**Published:** 2018-09-25

**Authors:** Joyce S. F. D. de Almeida, Rafael Dolezal, Ondrej Krejcar, Kamil Kuca, Kamil Musilek, Daniel Jun, Tanos C. C. França

**Affiliations:** 1 Laboratory of Molecular Modeling Applied to Chemical and Biological Defense, Military Institute of Engineering, Praca General Tiburcio 80, Rio de Janeiro 22290-270, Brazil; joycesfdalmeida@gmail.com; 2 Department of Chemistry, Faculty of Science, University of Hradec Kralove, Rokitanskeho 62, 50003 Hradec Kralové, Czech Republic; rafael.dolezal@fnhk.cz (R.D.); kamil.musilek@uhk.cz (K.M.); 3 Center for Basic and Applied Research, Faculty of Informatics and Management, University of Hradec Kralove, Rokitanskeho 62, 50003 Hradec Kralové, Czech Republic; ondrej.krejcar@uhk.cz; 4 Department of Toxicology, Faculty of Military Healthy Sciences, University of Defense, Trebesska 1575, 50001 Hradec Kralové, Czech Republic; daniel.jun@unob.cz

**Keywords:** aflatoxin B1, metabolites, acetylcholinesterase, catalytic anionic site

## Abstract

The most common type of aflatoxin (AFT) found in nature is aflatoxin B1 (AFB1). This micotoxin is extremely hepatotoxic and carcinogenic to mammals, with acute and chronic effects. It is believed that this could be related to the capacity of AFB1 and its metabolites in inhibiting the enzyme acetylcholinesterase (AChE). In a previous work, we performed an inedited theoretical investigation on the binding modes of these molecules on the peripheral anionic site (PAS) of human AChE (*Hss*AChE), revealing that the metabolites can also bind in the PAS in the same way as AFB1. Here, we investigated the binding modes of these compounds on the catalytic anionic site (CAS) of *Hss*AChE to compare the affinity of the metabolites for both binding sites as well as verify which is the preferential one. Our results corroborated with experimental studies pointing to AFB1 and its metabolites as mixed-type inhibitors, and pointed to the residues relevant for the stabilization of these compounds on the CAS of *Hss*AChE.

## 1. Introduction

In the beginning of the 1960s, contaminations of animal food with aflatoxins (AFT) were responsible for the death of turkeys, poults and pheasants in Great Britain, as well as adverse effects on ducks in South Africa, and trouts in the United States [1]. These mycotoxins are produced by the fungi of the genus *Aspergillus* (*A. flavus* and *A. parasiticus*) when they grow over grains in general, and dairy products, representing a serious problem for the storage and commercialization of agricultural commodities worldwide [2].

**Figure 1:**
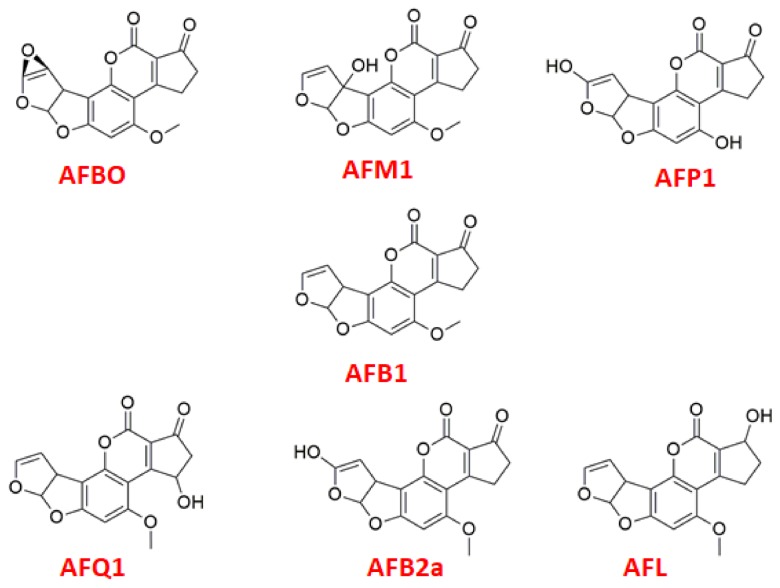
AFB1 and its metabolites.

**Figure 2:**
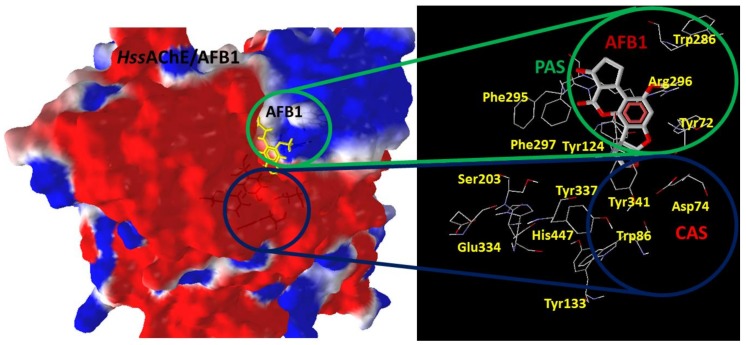
Model of *Hss*AChE used in this study with PAS and CAS highlighted in the green and blue circles, respectively.

**Figure 3:**
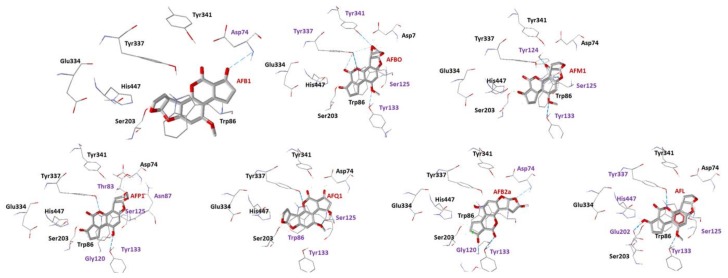
Best docking poses for the ligands on the CAS of *Hss*AChE. Residues involved in H-bonds are highlighted in purple.

AFT B1 (AFB1) is the most common type of AFT and figures amongst the most carcinogenic chemicals found in nature [3]. It can follow distinct metabolic routes, depending on the species contaminated, with four possible routes in humans [4]: (1) hydroxylation, to produce the metabolites AFB2a, AFP1, AFM1, and AFQ1 (Figure 1); (2) ketoreduction, to produce the metabolite AFL (Figure 1); (3) epoxidation, to produce 8,9-epoxide (AFBO) (Figure 1); and (4) o-dealkylation, to produce AFP1. AFBO is the most toxic and unstable of these metabolites. It can be easily converted to formamidopyrimidine, in physiologic environment, due to the opening of its epoxide ring, and interact with DNA, forming an AFB-N^7^-guaninine adduct, and triggering cancer and other damages to the liver [5,6,7].

Literature reports that AFB1 is capable of inhibiting the enzyme acetylcholinesterase (AChE) [8], and act as a neurotoxic compound [9], turning this toxin into a potential warfare agent. As AChE can be found mainly in neuromuscular junctions, and in the central and peripheral nervous systems (CNS and PNS), AFTs may interact with it at these sites due to its high hydrophobicity. These interactions could also be related to the carcinogenicity of AFB1 [10] and its metabolites.

It is known that AFB1, and probably also its metabolites, can bind to the peripheral anionic site (PAS) or the catalytic anionic site (CAS) of AChE [11]. The potential interactions of these compounds with the PAS of human AChE (*Hss*AChE) were theoretically investigated by our research group in a former work [12]. Here, we performed a similar investigation to verify if AFB1 and its metabolites can also bind favorably to the CAS of *Hss*AChE, and elucidate which residues are responsible for their stabilization. In addition, a comparison between the results obtained in both sites (PAS and CAS) was done to indicate the preferential one. We believe that this work will contribute to obtain more information about the preferential type of inhibition of AFB1, and its metabolites on *Hss*AChE, while helping to collect valuable information that will guide the design of new antidotes against AFT intoxication.

## 2. Results and Discussion

### 2.1. Docking Studies

As shown in Figure 2, the CAS of *Hss*AChE is located close to residues Asp74, Trp86, Tyr337 and Tyr341, while the PAS is close to residues Tyr72, Trp286 and Arg296. Most of CAS presents a low electrostatic potential and a hydrophilic solvent accessible surface, as shown in Figures S1 and S2. The results of our docking studies of AFB1 and its metabolites inside the CAS, in comparison to results obtained before for the PAS [12], are presented in Table 1.

The negative values of interacting energy observed for AFB1 and its metabolites (Table 1) suggest that they have affinity for the CAS. The metabolites AFP1 and AFQ1 presented the lowest, and very close to each other, intermolecular energy values, which suggest a better stabilization than the other metabolites. The residues Asp74, Thr83, Asn87, Gly120, Ser125, Tyr133 and Tyr337 contributed to the stabilization of AFP1 and Trp86, and Ser125 and Tyr337 to the stabilization of AFQ1. It is important to notice that Trp86 also performs hydrophobic interactions (π-stacking) with AFQ1, increasing its affinity for the CAS. Asp74, Ser125 and Tyr337 performed H-bond interactions with almost all metabolites, being important residues for their stabilization in the CAS. The best poses obtained for AFB1 and its metabolites are shown in Figure 3, while the 2D representations of their binding modes are shown in Figures S3–S9.

It is also important to notice that, for all metabolites, the binding energies on the CAS were lower than the values reported before for the PAS [12]. This suggests a higher affinity of these compounds for the CAS. Regarding the results for AFB1, the negative value of binding energy observed suggests affinity also for the CAS, which indicates the possibility of two binding modes of AFB1 inside *Hss*AChE: on the PAS, as shown before [12], and on the CAS, corroborating literature data suggesting that AFB1 can inhibit AChE in competitive and non-competitive ways [10,11].

### 2.2. Molecular Dynamics

**Figure 4:**
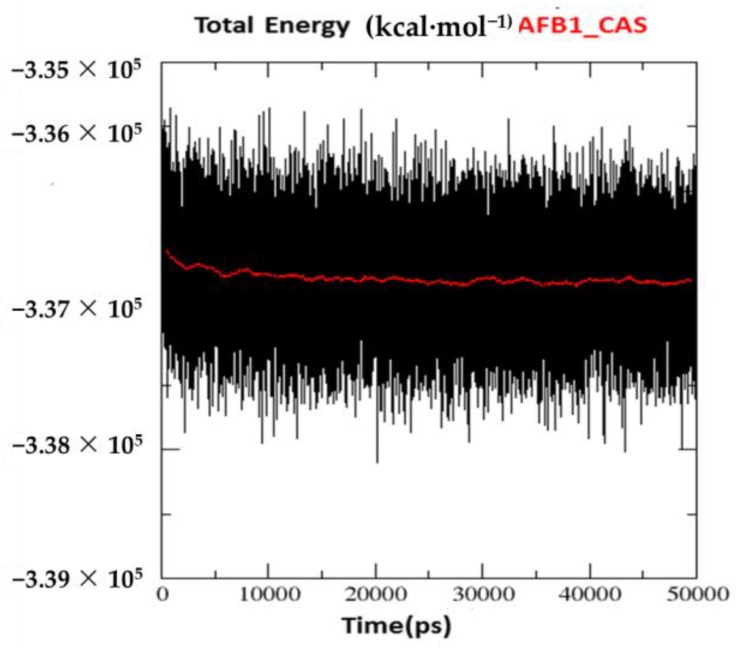
Variation of total energy for the complex *Hss*AChE/AFB1 on the CAS of *Hss*AChE.

The poses selected in docking studies were further submitted to MD simulations to check if they present stabilization on the chosen site during the simulation time. The resulting plots of energy variation through the simulated time, for the complex *Hss*AChE/AFB1, are shown in Figure 4, while the corresponding plots for the six *Hss*AChE/metabolite complexes are shown in Figure S10. These figures show a tendency to stabilization of the total energy after 2 ns for all systems, with an average value around −3.3 × 10^5^ kcal·mol^−1^.

**Figure 5:**
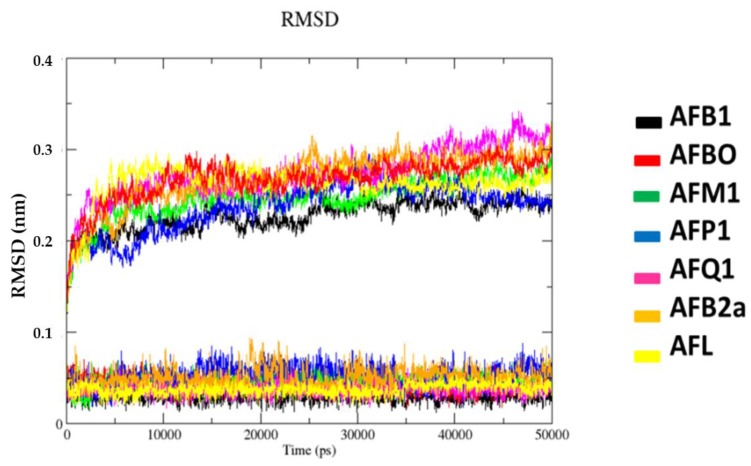
Variation of RMSD for the complexes studied.

Temporal RMSD plots shown in Figure 5 point to equilibrium after 2000 ps, for all systems, with deviations never passing 0.35 nm for protein and 0.1 nm for ligand. This suggests good accommodation of each ligand in the CAS during the simulated time.

RMSF plots shown in Figure S11 suggest that *Hss*AChE presented higher flexibility when complexed with AFBO and AFQ1, near Asp74 and Trp86, respectively. It is important to mention that these are the same residues observed in interactions with those metabolites in the docking studies.

**Figure 6:**
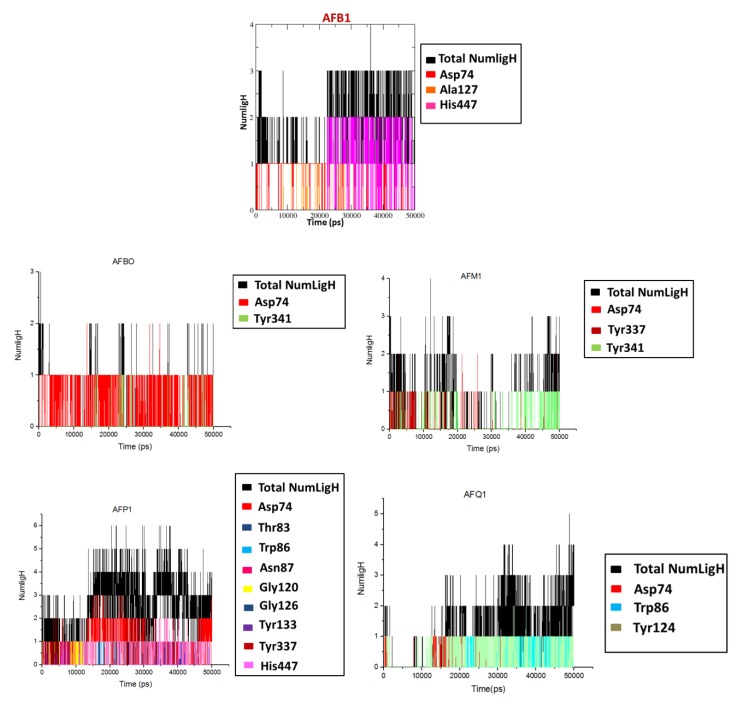

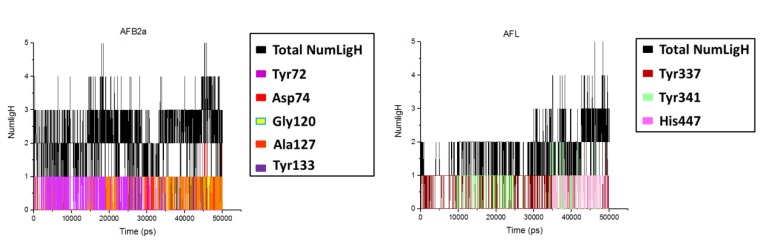
H-bonds observed for the complexes during the MD simulations.

Plots of H-bond interactions observed for the complexes during the simulated time are shown in Figure 6, while a comparison with the docking results is shown in Table 2. As can be seen, except for AFM1 and AFQ1, at least one H-bond was observed for each ligand in both studies.

**Figure 7:**
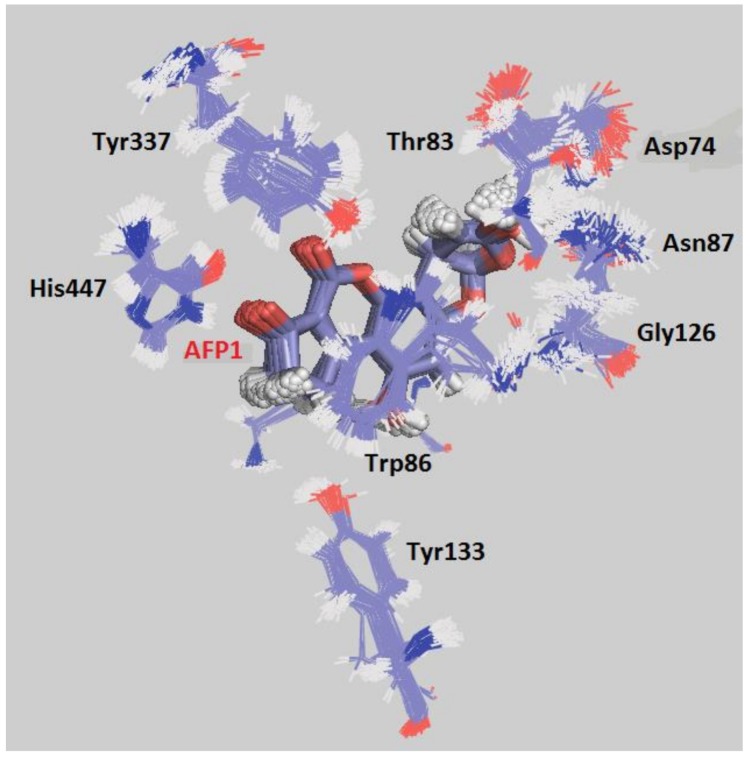
Superposition of frames for the complex *Hss*AChE/AFP1 during the MD simulation.

Superposition of MD frames during the MD simulations corroborate the stability of residues and ligands for all systems. Figure 7 shows the superposition of frames for the system *Hss*AChE/AFP1, while Figures S11–S16 show the same results for the other systems. For AFP1, besides the H-bond interaction with Trp86, the picture also shows a hydrophobic interaction (π-stacking) with this residue, which can contribute to increase the affinity of this metabolite for CAS.

### 2.3. MM-PBSA Calculations

**Figure 8:**
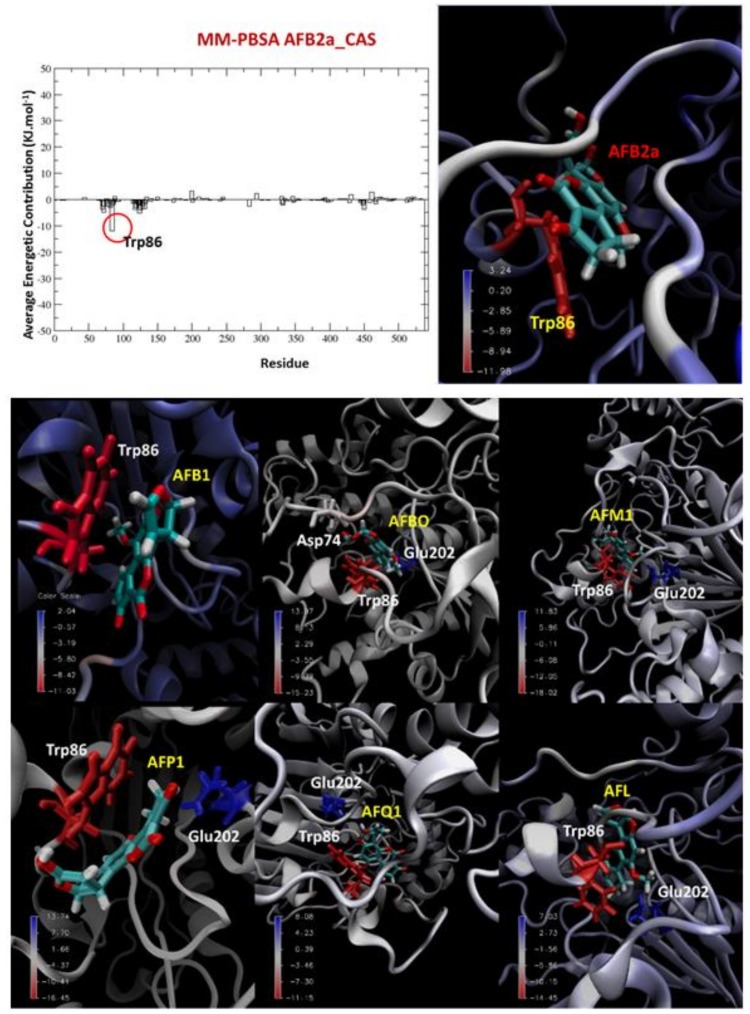
Illustration of the binding energy contributions for the complexes.

The MMPBSA results shown in Table 3 suggest that the Van Der Waals energy represents the higher contribution to stabilize the complexes, followed by the apolar (hydrophobic) solvation energy that is also negative. On the other hand, the polar (hydrophilic) solvation energy is positive, thus contributing to destabilize the systems. This result confirms the hydrophobic nature of AFB1 and its metabolites. Additionally, Table 4 shows that Trp86 was found to contribute favorably for all ligands, while Glu202 presents an unfavorable contribution, except in cases of AFB1 and AFB2a, the latter also presenting the best MM-PBSA binding energy. Illustrations of the energetic contributions for each ligand are shown in Figure 8. 

## 3. Conclusions

From the prediction of the solvent accessible surface area, we can infer that the CAS presents both hydrophilic and hydrophobic regions. It is expected that AFB1 and its metabolites would bind close to these hydrophobic parts, as observed in the molecular docking results. The energies observed were lower than in the PAS for all metabolites, suggesting that they may preferentially bind to the CAS and come closer to the active site. One example is the interaction of AFL with His447, which is part of the catalytic triad. Asp74, Trp86, Ser125 and Tyr337 were the residues contributing to stabilization in most systems. Regarding AFB1, our results suggest that it may present two binding modes: on PAS, as a non-competitive inhibitor, and on CAS, coming close to active site, and acting as a competitive inhibitor. According to the energy and RMSD plots obtained from the MD studies, it is possible to observe that all complexes tend to stabilization. The metabolites AFP1 and AFQ1 presented similar results and the highest affinities for the CAS, according to the docking and MD studies. As in docking studies, H-bonds with residues Asp74 and Tyr337 mainly contributed for the stabilization of the systems. MM-PBSA calculations presented very similar results for all metabolites, although AFB2a and AFB1 were slightly better than the others with no unfavorable interaction with any residue. Nevertheless, AFQ1 and AFP1 also presented good results in MM-PBSA studies. Van Der Waals/electrostatic and apolar contributions to the energy were negative and favored the systems. On the other hand, the polar solvation energy was positive and contributed to disfavor the complexes. This is expected, since AFB1 and its metabolites are highly hydrophobic compounds. In summary, our results suggest that the CAS is the preferential binding site for all the metabolites, being AFP1 and AFQ1 the best inhibitors of *Hss*AChE on this site.

## 4. Materials and Methods

### 4.1. Docking Energy Calculations

The model of the complex *Hss*AChE/AFB1 used in this work was obtained and validated as described before [12], through the PDB structures 2XI4 [13] and 4EY4 [14] and the software PC Spartan (Version Pro^®^, Wavefunction, Irvine, CA, USA, 1999) [15] and RM1 [16]. Figure 2 shows the 3D structure of the model, highlighting the positions of the PAS and the CAS.

Docking studies for the ligands on CAS of *Hss*AChE were performed with the software Molegro Virtual Docker (MVD)^®^ [17] (Version 6.0, CLC bio, Aarhus, Denmark, 2013), according to the same methodology used before [12], with a 13 Å radius sphere as binding site, and residues at 6 Å from the ligands treated as flexible. After each run, an automatic re-ranking score procedure through the algorithm Moldock score, was performed to assure the best configurations. For all compounds, 10 runs were performed with variations on initial population and maximum of interactions between them. For each run, 30 poses were obtained. Then, 300 poses for AFB1 and each metabolite were analyzed. The best conformations of each ligand, considering lower binding and H-bond energies, were chosen for further MD simulations and MM-PBSA calculations. The 2D representations of each pose selected were generated in the server LigPlot+ (Version 2.1, EMBL-EBI, Hinxton, Cambridgeshire, UK, 2015) [18].

### 4.2. Molecular Dynamics and Free Energy Calculations

Poses from the docking studies were prepared for the forcefield OPLS/AA [18] from the program GROMACS [19,20] (Version 5.1.4, Stockholm University and KTH, Stockholm, Sweden; Biomedical Centre, Uppsala, Sweden; Science for Life Laboratory and KTH, Stockholm, Sweden, 2016), following the same procedure as before [12], using ACPYPE [21,22] and MKTOP [23]. The complexes *Hss*AChE/ligand were simulated through GROMACS 5.1.4 [19,20] according to the same protocol used before [12,24], and results were analyzed with the software VMD [25] (Version 1.9.3, NIH resource for Macromolecular Modeling and Bioinformatics, University of Illinois, Urbana-Champaign, IL, USA, 2016), Grace (http://plasma-gate.weizmann.ac.il/Grace/) (Version 5.1.25, Grace Development team, Weizmann Institute of Science, Baden, Austria, 1995–2015) and PyMOL [26] (Version 1.7.x, Schrödinger LCC, New York, NY, USA, 2009–2014).

As before [12], the MM-PBSA [27] calculations were accessed through the s_mmpbsa tool [27] from the GROMACS package [19,20].

## Figures and Tables

**Table 1:** Docking results obtained for the ligands on the CAS of *Hss*AChE.

Ligand	E_Interaction_ (kcal·mol^−1^) CAS	H-Bond Interactions CAS	E_H-Bond_ (kcal·mol^−1^) CAS	E_Interaction_ (kcal·mol^−1^) PAS [12]
AFB1	−141.95	Asp74	−0.77	−56.68
AFBO	−137.73	Ser125 Tyr133 Tyr337 Tyr341	−8.82	−107.68
AFM1	−140.00	Tyr124 Ser125 Tyr133	−8.63	−117.75
AFP1	−146.87	Asp74 Thr83 Asn87 Gly120 Ser125 Tyr133 Tyr337	−15.21	−96.47
AFQ1	−146.17	Trp86 Ser125 Tyr337	−7.09	−104.67
AFB2a	−140.39	Asp74 Gly120 Tyr133	−3.48	−94.32
AFL	−137.98	Ser125 Tyr133 Glu202 Tyr337 His447	−7.60	−102.69

**Table 2:** Comparison of H-bonds observed in the docking and MD simulations for each ligand.

Ligand	Number of H-Bonds (Average)	H-Bond Observed during MD Simulations	Average Distance between Mass Centers (nm)	H-Bonds Observed in the Docking Studies
AFB1	3	Asp74 Ala127 His447	--	Asp74
AFBO	1	Asp74 Tyr341	0.86, 1.14	Tyr133 Tyr337, Tyr341
AFM1	2	Asp74 Tyr337 Tyr341	0.91, 0.83, 0.77	Tyr124 Ser125 Tyr133
AFP1	5	Asp74 Thr83 Trp86 Asn87 Gly120 Gly126 Tyr133 Tyr337 His447	0.84, 0.74, 0.40 0.78, 0.80, 0.67 1.07, 0.92, 0.91	Asp74 Thr83 Asn87 Gly120 Ser125 Tyr133 Tyr337
AFQ1	3	Asp74 Trp86 Tyr124	0.93, 0.49, 0.70	Trp86 Ser125 Tyr337
AFB2a	3	Tyr72 Asp74 Gly120 Ser125 Ala127 Tyr133	1.04, 0.99, 0.68, 0.62, 0.74, 1.02	Asp74 Gly120 Tyr133
AFL	3	Tyr337 Tyr341His447	1.02, 0.84, 0.97	Ser125 Tyr133 Tyr337

**Table 3:** Energetic contributions from MMPBSA for the complexes.

Ligand	MM-PBSA Average Binding Energy (kcal·mol^−1^)	Van Der Waals/Electrostatic Energy (kcal·mol^−1^)	Polar Solvation Energy (kcal·mol^−1^)	Apolar Solvation Energy (kcal·mol^−1^)	Molecular Docking Energy (kcal·mol^−1^)
AFB1	−29.23	−47.35	22.14	4.03	−141.95
AFBO	−26.82	−42.91	19.78	−3.69	−137.73
AFM1	−26.11	−48.42	26.65	−4.35	−140.00
AFP1	−27.93	−51.32	27.35	−3.96	−144.50
AFQ1	−26.67	−47.57	25.04	−4.14	−146.17
AFB2a	−32.36	−59.09	30.83	−4.09	−140.39
AFL	−26.28	−47.83	25.67	−4.12	−137.98

**Table 4:** Energetic contributions of for the complexes *Hss*AChE/ligands.

Ligand	Favorable Energetic Contributions	Unfavorable Energetic Contributions
AFB1	Trp86	-
AFBO	Trp86 Asp74	Glu202
AFM1	Trp86	Glu202
AFP1	Trp86	Glu202
AFQ1	Trp86	Glu202
AFB2a	Trp86	-
AFL	Trp86	Glu202

## Supplementary Materials

The following are available online at http://www.mdpi.com/2072-6651/10/10/389/s1, Figure S1: Map of electrostatic potential for the CAS. Regions of higher electrostatic potentials are represented in blue while lower electrostatic potentials are in red; Figure S2: Solvent accessible surface for the protein. Hydrofilic areas are represented in red and hydrophobic areas are represented in blue; Figure S3: 2D representation of the best pose of AFB1 on CAS; Figure S4: 2D representation of the best pose of AFBO on CAS; Figure S5: 2D representation of the best pose of AFM1 on CAS; Figure S6: 2D representation of the best pose of AFP1 on CAS; Figure S7: 2D representation of the best pose of AFQ1 on CAS; Figure S8: 2D representation of the best pose of AFB2a on CAS; Figure S9: 2D representation of the best pose of AFL on CAS; Figure S10: Variation of total energy for the complexes HssAChE/AFB1-metabolites on the CAS of HssAChE; Figure S11: Superposition of frames for the complex HssAChE/AFB1 on the CAS during 50 ns of MD simulations; Figure S12: Superposition of frames for the complex HssAChE/AFBO on the CAS during 50 ns of MD simulations; Figure S13: Superposition of frames for the complex HssAChE/AFM1 on the CAS during 50 ns of MD simulations; Figure S14: Superposition of frames for the complex HssAChE/AFQ1 on the CAS during 50 ns of MD simulations; Figure S15: Superposition of frames for the complex HssAChE/AFB2a on the CAS during 50 ns of MD simulations; Figure S16: Superposition of frames for the complex HssAChE/AFL on the CAS during 50 ns of MD simulations.
