# Supplementary material for: Molecular Modeling Studies on the Interactions of Aflatoxin B1 and Its Metabolites with Human Acetylcholinesterase. Part II: Interactions with the Catalytic Anionic Site (CAS)
Source: Toxins (Basel). 2018 Sep 25;10(10):389. doi: 10.3390/toxins10100389 (PMC6215247; doi:10.3390/toxins10100389)
Supplement: Supplementary file 1 [file toxins-10-00389-s001.pdf]

# Supplementary Materials: Molecular Modeling Studies on the Interactions of Aflatoxin B1 and Its Metabolites with Human Acetylcholinesterase. Part II: Interactions with the Catalytic Anionic Site (CAS)

**Joyce S. F. D. de Almeida, Rafael Dolezal, Ondrej Krejcar, Kamil Kuca, Kamil Musilek, Daniel Jun and Tanos C. C. França**

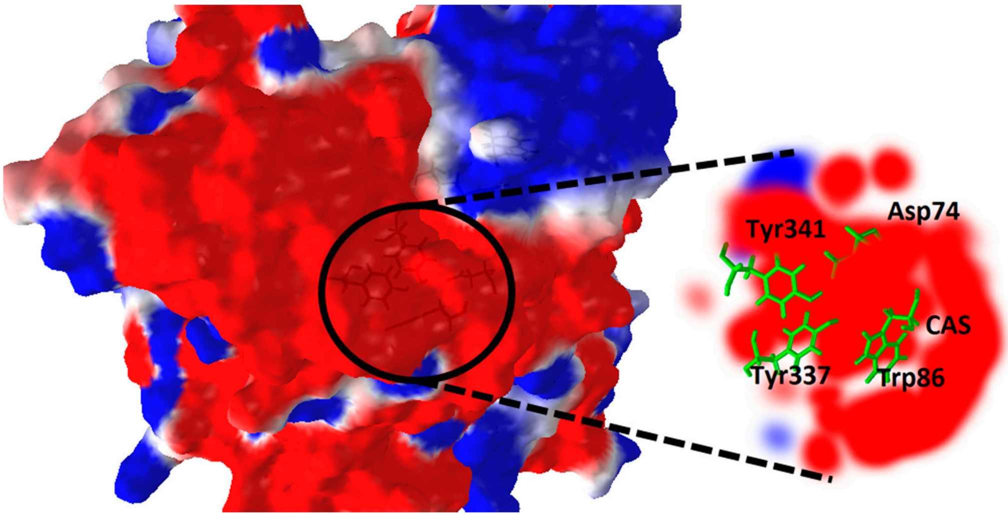


**Figure S1.** Map of electrostatic potential for the CAS. Regions of higher electrostatic potentials are represented in blue while lower electrostatic potentials are in red.

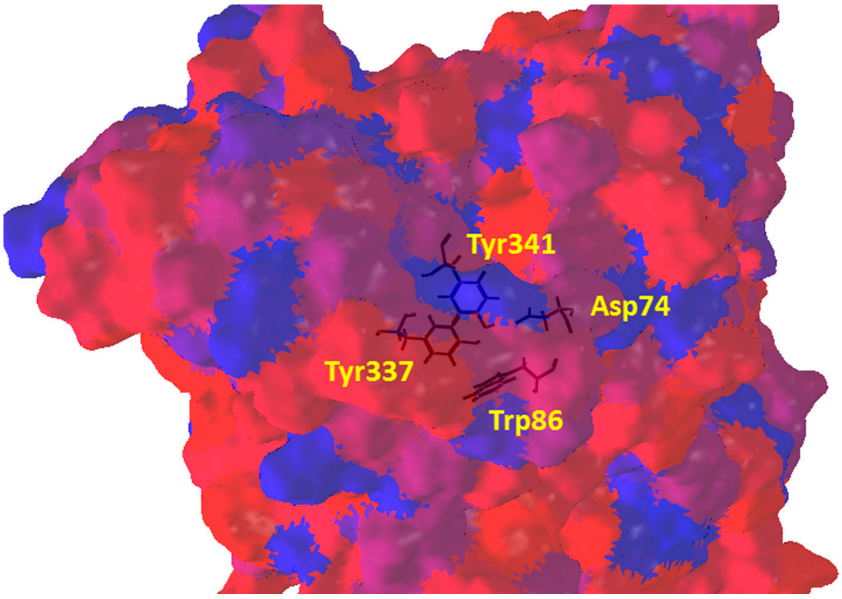


**Figure S2.** Solvent accessible surface for the protein. Hydrofilic areas are represented in red and hydrophobic areas are represented in blue.

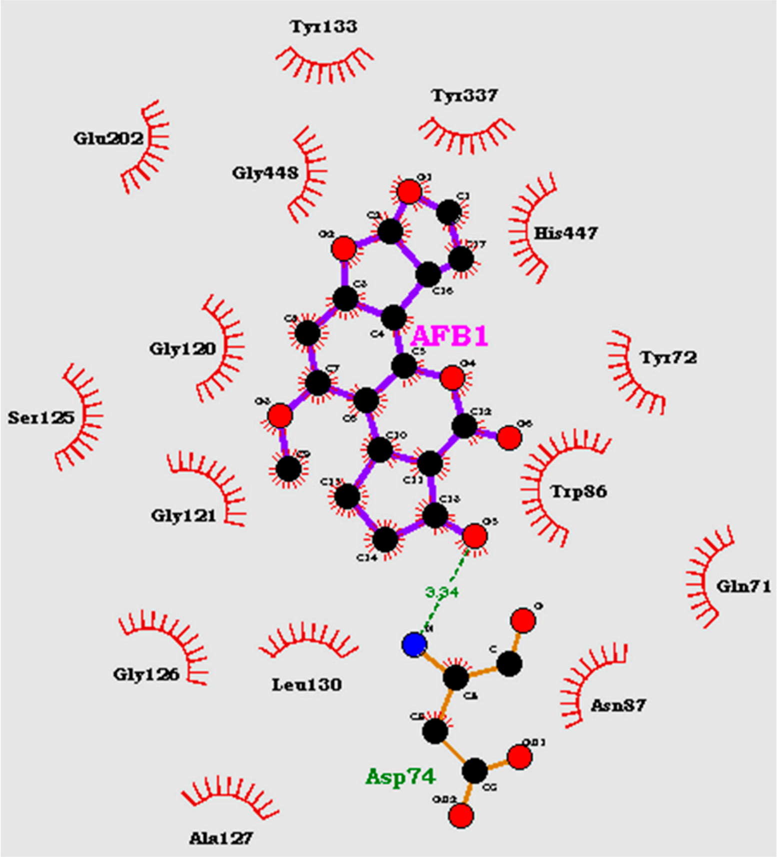


**Figure S3.** 2D representation of the best pose of AFB1 on CAS.

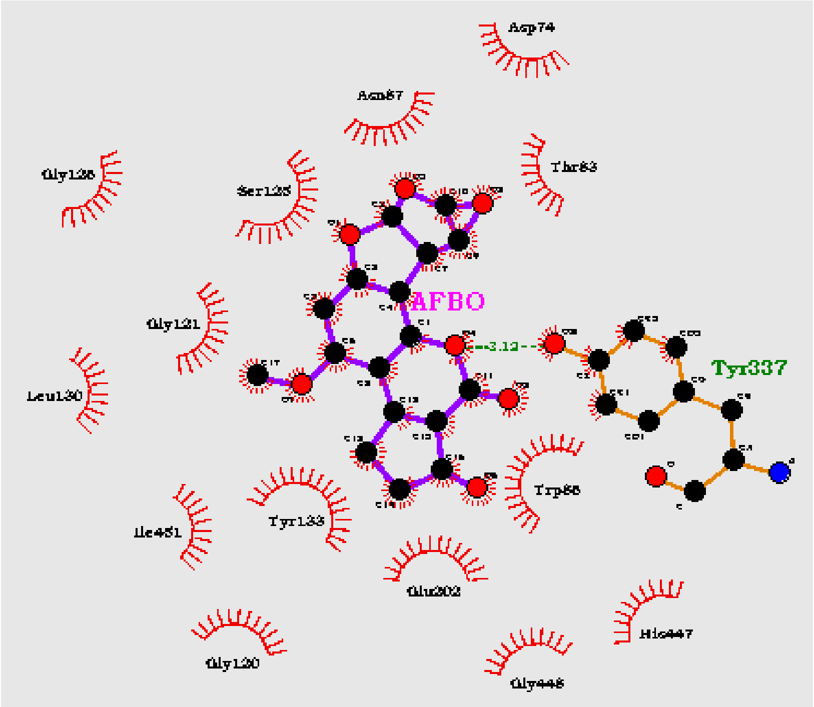


**Figure S4.** 2D representation of the best pose of AFBO on CAS.

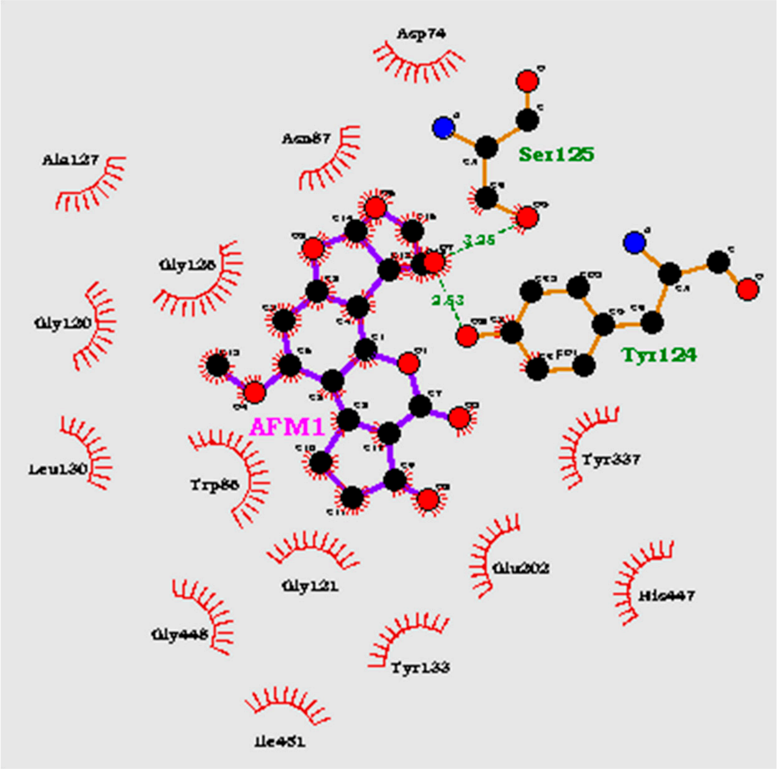


**Figure S5.** 2D representation of the best pose of AFM1 on CAS.

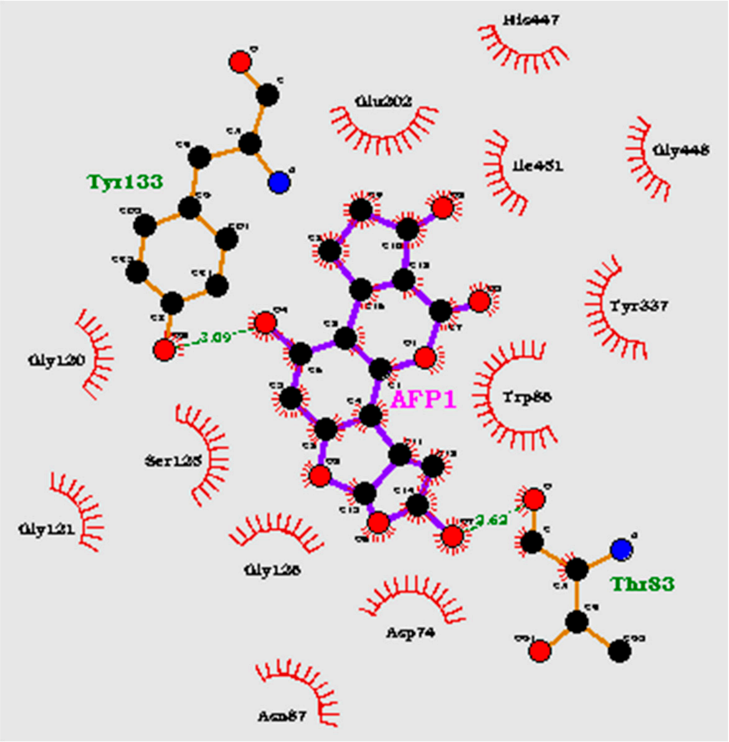


**Figure S6.** 2D representation of the best pose of AFP1 on CAS.

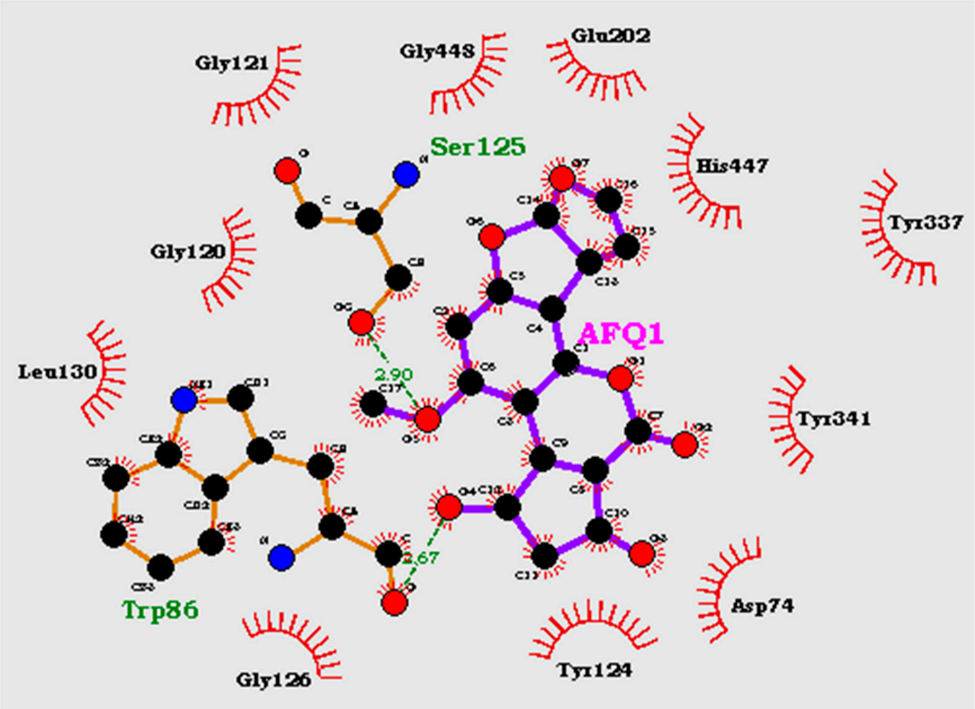


**Figure S7.** 2D representation of the best pose of AFQ1 on CAS.

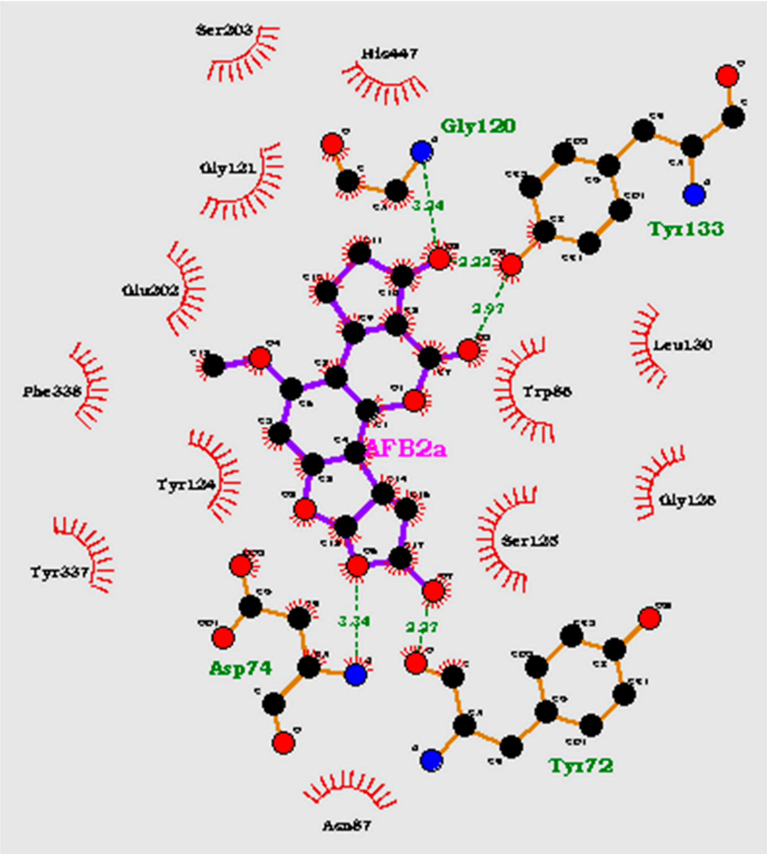


**Figure S8.** 2D representation of the best pose of AFB2a on CAS.

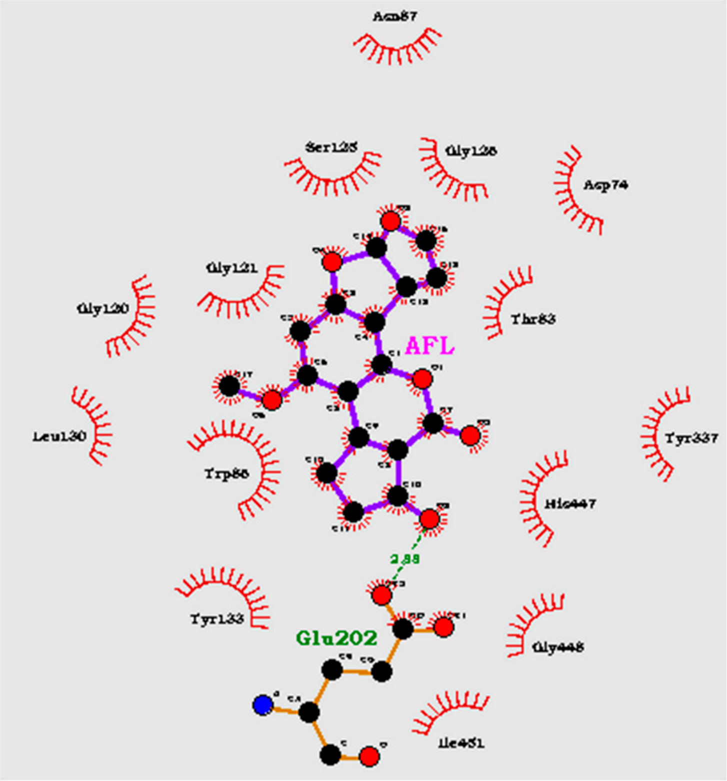


**Figure S9.** 2D representation of the best pose of AFL on CAS.

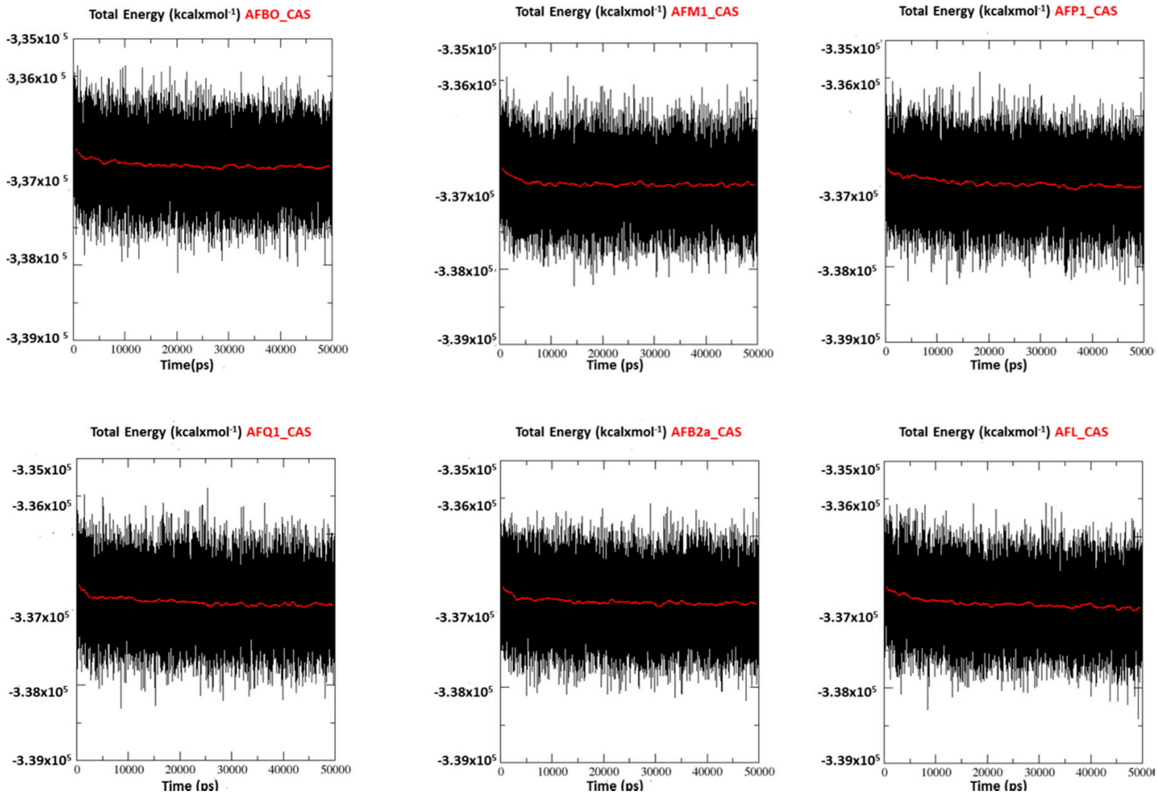


**Figure S10.** Variation of total energy for the complexes *Hss*AChE/AFB1-metabolites on the CAS of *Hss*AChE.

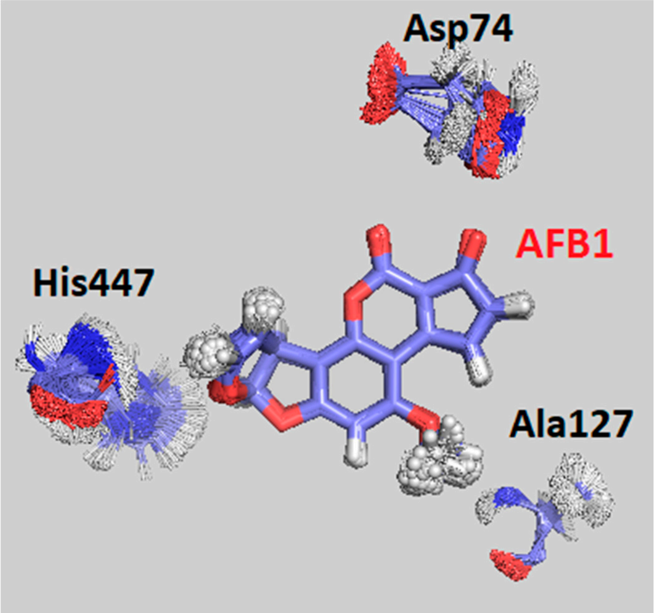


**Figure S11.** Superposition of frames for the complex *Hss*AChE/AFB1 on the CAS during 50 ns of MD simulations.

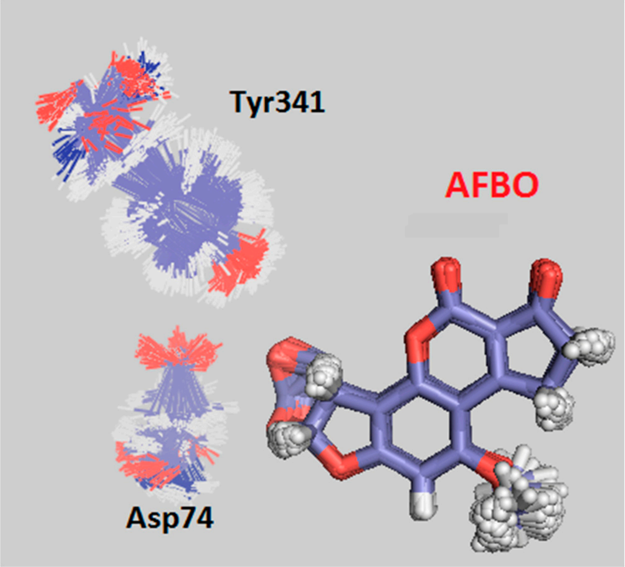


**Figure S12.** Superposition of frames for the complex *Hss*AChE/AFBO on the CAS during 50 ns of MD simulations.

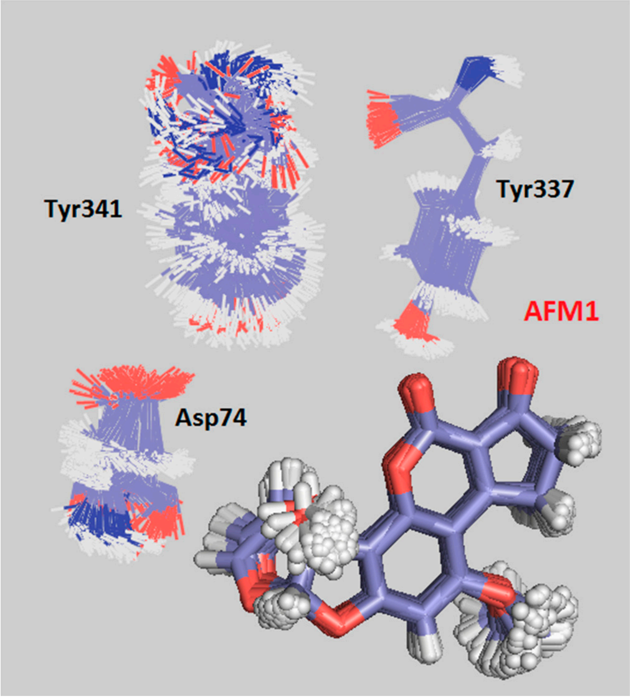


**Figure S13.** Superposition of frames for the complex *Hss*AChE/AFM1 on the CAS during 50 ns of MD simulations.

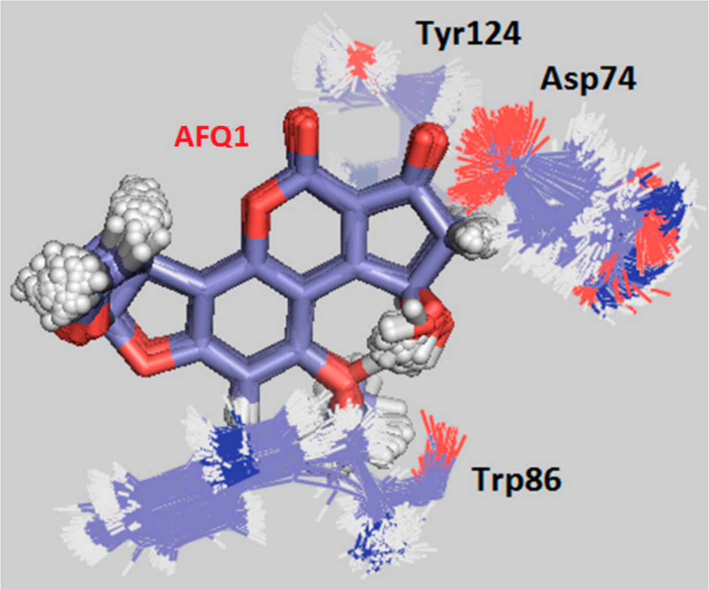


**Figure S14.** Superposition of frames for the complex *Hss*AChE/AFQ1 on the CAS during 50 ns of MD simulations.

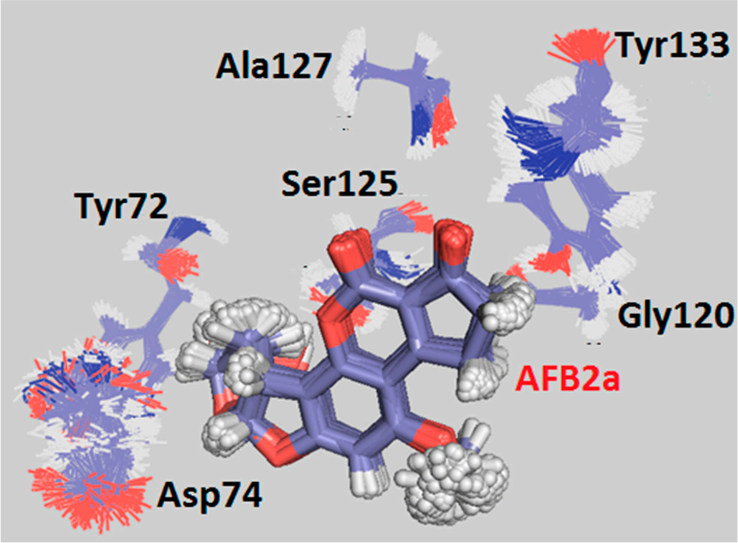


**Figure S15.** Superposition of frames for the complex *Hss*AChE/AFB2a on the CAS during 50 ns of MD simulations.

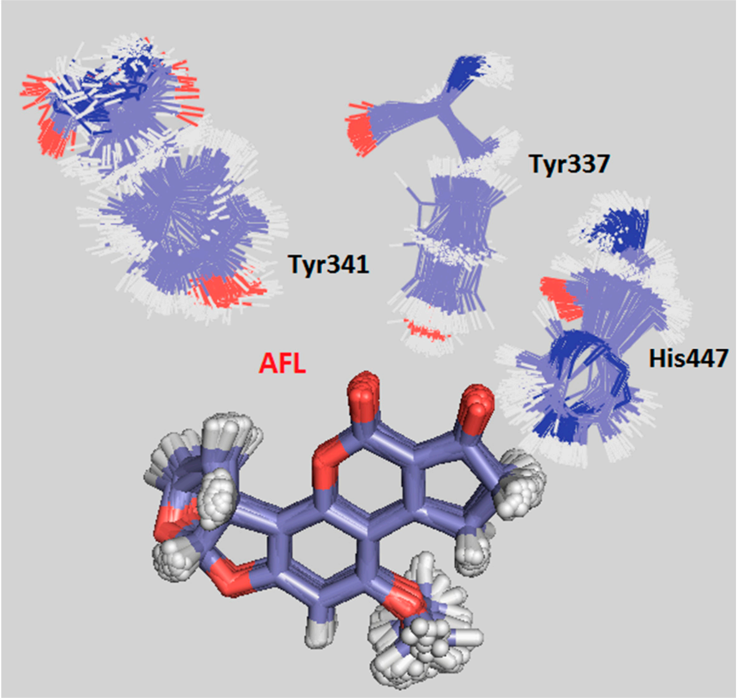


**Figure S16.** Superposition of frames for the complex *Hss*AChE/AFL on the CAS during 50 ns of MD simulations.
